# Ultrasound-Triggered Phase Transition Sensitive Magnetic Fluorescent Nanodroplets as a Multimodal Imaging Contrast Agent in Rat and Mouse Model

**DOI:** 10.1371/journal.pone.0085003

**Published:** 2013-12-31

**Authors:** Xin Cheng, Huan Li, Yunchao Chen, Binhua Luo, Xuhan Liu, Wei Liu, Haibo Xu, Xiangliang Yang

**Affiliations:** 1 College of Life Science and Technology, Huazhong University of Science and Technology, Wuhan, Hubei, China; 2 National Engineering Research Center for Nanomedicine, Huazhong University of Science and Technology, Wuhan, Hubei, China; 3 College of Pharmacy, Yunnan University of Traditional Chinese Medicine, Kunming, Yunnan, China; 4 Department of Radiology, Union Hospital, Tongji Medical College, Huazhong University of Science and Technology, Wuhan, Hubei, China; 5 Department of Medical Ultrasound, Tongji Hospital, Tongji Medical College, Huazhong University of Science and Technology, Wuhan, Hubei, China; NIH, United States of America

## Abstract

Ultrasound-triggered phase transition sensitive nanodroplets with multimodal imaging functionality were prepared *via* premix Shirasu porous glass (SPG) membrane emulsification method. The nanodroplets with fluorescence dye DiR and SPIO nanoparticles (DiR-SPIO-NDs) had a polymer shell and a liquid perfluoropentane (PFP) core. The as-formed DiR-SPIO-NDs have a uniform size of 385±5.0 nm with PDI of 0.169±0.011. The TEM and microscopy imaging showed that the DiR-SPIO-NDs existed as core-shell spheres, and DiR and SPIO nanoparticles dispersed in the shell or core. The MTT and hemolysis studies demonstrated that the nanodroplets were biocompatible and safe. Moreover, the proposed nanodroplets exhibited significant ultrasound-triggered phase transition property under clinical diagnostic ultrasound irradiation due to the vaporization of PFP inside. Meanwhile, the high stability and R_2_ relaxivity of the DiR-SPIO-NDs suggested its applicability in MRI. The *in vivo* T_2_-weighted images of MRI and fluorescence images both showed that the image contrast in liver and spleen of rats and mice model were enhanced after the intravenous injection of DiR-SPIO-NDs. Furthermore, the ultrasound imaging (US) in mice tumor as well as MRI and fluorescence imaging in liver of rats and mice showed that the DiR-SPIO-NDs had long-lasting contrast ability *in vivo*. These *in vitro* and *in vivo* findings suggested that DiR-SPIO-NDs could potentially be a great MRI/US/fluorescence multimodal imaging contrast agent in the diagnosis of liver tissue diseases.

## Introduction

Multimodal imaging has played an ever-increasing role in the diagnosis and prognosis of liver tissue diseases [1]. To date, a number of noninvasive, quantitative, functional imaging techniques are currently used in standard clinical practice, such as magnetic resonance imaging (MRI), ultrasound imaging (US), and optical imaging (fluorescence imaging) [2]. However, each imaging modality has its particular advantages and disadvantages. For example, MRI is a soft-tissue contrast imaging modality with high spatial resolution and multi-planar imaging capacities, but its cost is relatively high and the imaging time is long with relatively low sensitivity. US is a real-time, low cost, non-ionizing and widely available imaging tool, but its resolution is low and largely depends on the analysis of operator. Fluorescence imaging is an imaging modality with high sensitivity and multicolor, but it is non-quantitative with poor tissue penetrating ability. Therefore, different imaging modalities are generally considered as complementary rather than competitive [3], [4]. To utilize the strengths of different imaging methods, multimodality imaging has become an attractive strategy for clinical research. The integration of several imaging contrast agents with different capabilities in multifunctional nanoparticles would obtain more accurate and reliable information about diseases through combining multimodal imaging [5].

Recently, multimodal imaging has become the frontier technology by maximizing the advantages of nano contrast agents, and some groups have put great efforts on the development of dual-modal and triple-modal imaging [6]–[9]. In these studies, some microbubbles contrast agents including magnetic nanoparticles or fluorescent dyes were applied to MRI/US or US/fluorescence imaging [7], [10]. Dai group has successfully constructed quantum dots-modified microbubbles and graphene oxide modified microcapsules for US/fluorescent and US/CT bimodal imaging, respectively [11], [12]. Despite great progress has been acquired in multimodal imaging field, microbubbles are still considered as blood-pool agents due to their large size, because they couldn’t penetrate the gaps between tumor vascular endothelial cells. To realize extravascular imaging, nanobubbles (nano-sized contrast agents) can be applied for the site-specific delivery of these contrast agents. For instance, Zheng *et al.* reported the red fluorescence-dyed nanobubbles and Mai *et al.* reported cyanine 5.5 conjugated nanobubbles had achieved tumor-selective imaging due to the enhanced permeation and retention (EPR) effect at tumor vascular leaks [13], [14].

However, to date, the research on nanobubbles is still in the initial stages. The commonly used preparation methods of nanobubbles are similar as microbubbles, such as probe-type sonication method [14], thin-film hydration-sonication method [15], double emulsion freeze-drying method [8], and mechanical agitation method [16]. Although great progress has been achieved in this field, some major limitations of these nano/microbubbles, including inevitably relatively broad size distribution and hard to reproduce, remains to be investigated. Additionally, these conventional methods might cause loss of functional properties of shear and heat sensitive components due to strong mechanical action and high temperature. In recent years, the Shirasu porous glass (SPG) membrane emulsification technique has been developed rapidly because it provides a facile procedure with lower energy and narrow size distribution [17], [18]. For example, Ma and coworkers have prepared uniform-size polymer microcapsules and microspheres with excellent reproducibility *via* SPG membrane emulsification method [19], [20]. Moreover, size control of monodispersed nanobubbles were generated through SPG membranes by Kukizaki [21].

Nano/microbubbles are often composed of various shells (polymers, surfactants and lipids) and cores (gas, liquid). Among these materials, polymer-based hard-shell microbubbles is superior to surfactant/phospholipid-based soft-shell microbubbles, because the rigid shell give these microbubbles much more stability and longer circulation lifetime *in vivo* [16], [22]. For instance, the commercially available microbubbles Definity, with surfactant and lipid shell, provided only 3–5 min of ultrasound contrast after bolus injection [23]. Furthermore, it was reported that, with ultrasound irradiation at physiological temperature, nanodroplets containing perfluoropentane (PFP) could be turned to nanobubbles and microbubbles due to the low boiling point (29°C) of PFP at atmospheric pressure. The safety and ultrasound imaging capability of these nanobubbles were also proved [24]–[26].

**Figure 1:**
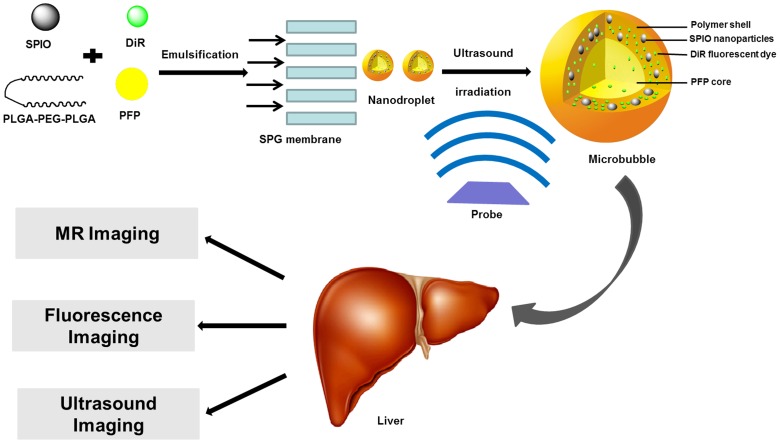
Schematic diagram of the designed ultrasound-triggered phase transition DiR-SPIO-NDs with multimodal imaging functionality.

In this study, we prepared uniform-size nanodroplets encapsulating both magnetic nanoparticles and fluorescent dyes *via* the premix SPG membrane emulsification method. The nanodroplets had a poly(lactic-co-glycolic acid) and poly(ethylene glycol) (PLGA-PEG-PLGA) shell and liquid PFP core. In particular, PLGA-PEG-PLGA was a biodegradable and biocompatible shell material, which ensured the safety for *in vivo* applications. The liquid PFP core enhanced the storage stability and empowered the nanodroplets a capability of ultrasound-triggered phase transition (Figure 1 and S1). Furthermore, the morphology characterization, ultrasound-triggered phase transition, biocompatibility, *in vitro* R_2_ relaxivity and the feasibility of these vehicle acoustic nanodroplets as multimodal imaging contrast agents for MRI/US/fluorescence imaging in rats and mice model were investigated.

## Materials and Methods

### 1. Materials

Copolymer from poly(lactic-co-glycolic acid) and poly(ethylene glycol) (PLGA-PEG-PLGA) was purchased from Shandong Institute of Medical Instrument (Shandong, China), with a PEG block molecular weight of 2000 Da and LA:GA = 50∶50 in the PLGA block. The mean molecular weight of PLGA-PEG-PLGA was 20,000 Da and the polydispersity (Mw/Mn) was 1.7. Poly(vinyl alcohol) (PVA-217, 1700 of polymerization degree, 88.5% of hydrolysis degree) was obtained from Kuraray (Tokyo, Japan). The near-infrared fluorescence dye 1,1′-dioctadecyltetramethyl indotricarbocyanine iodide (DiR) was purchased from Biotium (Hayward, CA, USA). Perfluoro-n-pentane (PFP) was obtained from Strem Chemicals (Newburyport, USA). SPG membrane (pore size of the membrane was 1 µm) was provided by SPG Technology Co. Ltd. (Japan). All other reagents were of analytical grade.

Superparamagnetic iron oxide (SPIO) nanoparticles (mean diameter in about 10 nm) were synthesized *via* the reductive decomposition of Fe(acac)_3_ in benzyl ether and oleylamine as reported by Sun *et al.* [27] with minor modification. Briefly, Fe(acac)_3_ (2 mmol) was dissolved in 7.5 mL of benzyl ether and 7.5 mL of oleylamine. The solution was dehydrated at 110°C under N_2_ atmosphere for 1 h, then quickly heated to 290°C at a heating rate of 15°C/min, aged at 290°C for another 1 h. After the solution was cooled down to room temperature, Fe_3_O_4_ nanoparticles were extracted with the addition of 50 mL of ethanol and collected through centrifuging. The SPIO nanoparticles were dispersed in tetrahydrofuran (THF).

### 2. Fabrication of Nanodroplets with Embedded DiR and SPIO

Nanodroplets with PVA outer layer and PLGA-PEG-PLGA inner layer were prepared *via* premix membrane emulsification with SPG membrane and the double emulsion solvent evaporation (gas-in-oil-in-water emulsion) process. Briefly, the coarse double emulsions were first prepared. The tetrahydrofuran solution (5 mL) containing DiR (1 mg), SPIO nanoparticles (50 mg) and PLGA-PEG-PLGA (0.1 g) was prepared, then PFP (1 mL) was added and homogenized with 10000 rpm for 2 min (FLUKO homogenizer, FA25 model) in an ice bath and in the dark to form primary emulsion. The primary emulsion was poured into 35 mL normal saline solution containing 0.3% PVA (w/v) and mixed mechanically in an ice bath and in the dark for 2 min to form coarse double emulsions. The coarse double emulsions were then poured into the reservoir of miniature kit for premix membrane emulsification (SPG techno fast mini kit KH-125), then double emulsions with smaller and relatively uniform size were achieved *via* extruding the coarse double emulsions through the SPG membrane with 97 psi of pressure five times at 25°C. Finally, the obtained double emulsions were poured into 100 mL normal saline solution and stirred overnight in an ice bath and in the dark to evaporate organic solvent and solidify the nanodroplets. The nanodroplet suspension was centrifuged (8228 g, 10 min) at 4°C (Eppendorf, Centrifuge 5810R) to obtain nanodroplet precipitate, and the precipitate was dispersed into normal saline solution with about 2×10^9^ nanobubbles/mL and stored in aluminum foil at 4°C for further studies. Nanodroplets (NDs) without DiR dye and SPIO nanoparticles were prepared through the same procedure. Figure 1 showed the designed nanodroplets with embedded DiR and SPIONs (DiR-SPIO-NDs).

The nanodroplet structure can be briefly described as the following: The gas core of the double emulsion was PFP (boiling point 29°C), which was encapsulated by the polymer shell of PLGA-PEG-PLGA and PVA. Hydrophobic SPIO nanoparticles were dispersed in the PLGA-PEG-PLGA layer, and DiR fluorescence dyes in the PLGA-PEG-PLGA and PFP layers.

### 3. Characterization of Nanodroplets

The hydrodynamic size, polydispersity distribution index (PDI) and zeta potential of both DiR-SPIO-NDs and NDs were measured on a dynamic light scattering instrument (DLS, Zetasizer Nano ZS90, Malvern Instruments Ltd., Worcestershire, UK) with a scattering angle of 90° at 37°C. In order to evaluate the stability of NDs, the size and PDI of NDs were analyzed in normal saline in 1, 2, 3, 4, 5, 6, 8, 14, 22, 30, 90, 108, 149, 183, 380 days, respectively.

The concentration of nanobubbles with embedded DiR and SPIONs (DiR-SPIO-NBs) was measured by a hemacytometer after DiR-SPIO-NDs with a 1000 folds dilution ratio were sonicated (frequency = 40 kHz, ultrasonic power = 100 W) by an ultrasonic cleaner (KQ-100, China) for 2 min at 37°C. The concentration was calculated using the similar cell-counting method. In order to directly visualize the nanobubbles (NBs), they were placed between glass slides to examine on an inverted fluorescence microscopy (Olympus IX71, Japan) with the 400× magnification in bright and fluorescence visual field.

Morphologies of DiR-SPIO-NDs and NDs were examined by a transmission electron microscopy (TEM, Tecnai G^2^ 20 TWIN, FEI Corp. USA) at 200 kV accelerating voltage. The samples were prepared through drying the dispersion on amorphous carbon coated copper grids and negative staining with 2% (w/v) sodium phosphotungstate solution.

Total iron content of DiR-SPIO-NDs emulsions were determined on a fast sequential atomic absorption spectrometer (Spectr AA-240FS, Varian, Palo Alto, USA).

### 4. Biocompatibility Tests

#### 4.1. Cell culture

Human hepatoma cell line HepG2 was obtained from ATCC (VA, USA). Human normal liver cell line HL-7702 was purchased from Shanghai Institute of Life Science Cell Culture Center (Shanghai, China). These cell lines were cultured in Dulbecco modified Eagle’s medium (DMEM) with 10% fetal calf serum (Gibco, USA), antibiotics penicillin (100 IU/mL) and streptomycin (100 mg/mL) in a humidified atmosphere with 5% CO_2_ at 37°C.

#### 4.2. Cytotoxicity assay

Cytotoxicity of the magnetic fluorescent nanodroplets was assessed by MTT assay. HepG2 cells and HL-7702 cells were used, respectively, to assess cell viability. Cells were seeded at a density of 8000 cells/well in a 96-well plate and incubated for 24 h. Then, the medium with DiR-SPIO-NDs was added in to dilute the cell medium until it contained 0, 0.5, 1, 2, 3, 4, 5, 6, 8 and 10% (v/v) of NDs, respectively. The control well was filled with only culture medium without DiR-SPIO-NDs. After 24 and 48 h of incubation, respectively, 20 µL of 3-(4,5-dimethylthiazol-2-yl)-2,5-diphenyltetrazolium bromide (MTT, 5 mg/mL) was added to wells. After 4 h of incubation, medium was removed and DMSO (150 µL/well) was added to dissolve the formazan crystals. The absorbance of each well was read on a microplate reader (318C-Microplate reader, China) at 492 nm. The relative cell viability (RCV) was calculated as the following equation:
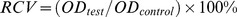
here, OD_test_ was obtained in the presence of DiR-SPIO-NDs and OD_control_ was obtained in the absence of DiR-SPIO-NDs.

#### 4.3. Hemolysis assay

SD rat blood was collected in a tube with heparin sodium (15 IU/mL) and centrifuged at 1500 rpm for 15 min to harvest erythrocytes. The cell pellet was washed three times with cold normal saline by centrifugation at 1500 rpm for 25 min and resuspended in normal saline to achieve a concentration of 2% (v/v). Within 24 h after the collection of erythrocytes, suspension was prepared and used. DiR-SPIO-NDs was diluted in normal saline to have different concentrations (the final suspension contained 1, 2, 3, 4, 5, 6, 8, 10% (v/v) of NDs, respectively). For each concentration, 150 µL of sample solution was mixed with 150 µL of 2% erythrocytes. The negative control (0% of hemolysis) and positive control (100% of hemolysis) were obtained by mixing 150 µL of normal saline or 150 µL of double-distilled water with 2% of erythrocytes, respectively. Then the resulting suspensions were incubated at 37°C for 1 h in a water bath, and centrifuged at 3000 rpm for 25 min. The supernatant was added to a 96-well plate and examined spectrophotometrically at 540 nm on a microplate reader. The hemolytic percentage was calculated by the following equation:
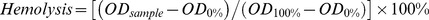
here, OD_sample_ was obtained in the presence of DiR-SPIO-NDs, OD_0%_ was obtained in the negative control and OD_100%_ was obtained in the positive control.

### 5. *In vitro* Imaging Study

#### 5.1. In vitro MRI

The MRI of DiR-SPIO-NDs *in vitro* study was performed at room temperature on a 3.0 Tesla whole-body MR scanner (MAGNETOM Trio, A Tim System 3T, Siemens, Munich, Germany) with a 12-channel head coil. To determine the relaxivity, the two samples (DiR-SPIO-NDs and hydrophilic SPIO nanoparticles (DMSA-coated Fe_3_O_4_ nanoparticles were from Nanjing Nanoeast Biotech Co., Ltd, China)) were diluted with normal saline to have iron concentrations of 0−0.0893 mmol/L. 0.5 mL Eppendorf tubes were filled with the samples and inserted in a 96-well plate, then the plate was placed in the MR scanner. T_2_-weighted images were acquired by using a spin echo sequence. The used parameters were as the following: Field of view (FOV) = 100 mm, averages = 3, base resolution = 128×128, slice thickness = 3 mm, multiple echo times (TE) = 40, 80, 120, 160, 200, 240 and 280 ms, repetition time (TR) = 2890 ms, and scanning time = 20–21 min. The T_2_ relaxation time was computed by using MATLAB V7.1. The R_2_ relaxation rates were plotted against the iron concentrations in the samples. The relaxivity was determined by a linear fit.

#### 5.2. In vitro ultrasound imaging

To prove the ultrasonic imaging ability of DiR-SPIO-NDs, compare with microbubbles (MBs), *in vitro* ultrasound imaging was carried out on a clinical ultrasonic scanner system (Logiq9, GE, USA) combined with a 3.5C abdominal transducer. A latex finger cot filled with degassed normal saline was placed into a 37°C water bath, and the sample (2 mL) was injected slowly into the latex finger cot, simultaneously, the probe was positioned below the water bath box. Three types of samples, including normal saline, DiR-SPIO-NDs (5.6×10^7^ nanobubbles/mL) and SonoVue (1.6×10^7^ microbubbles/mL) (commercially available microbubbles, Bracco imaging B. V., Switzerland), were used. The 3.5 MHz ultrasound transducer was used as a transmitter and a receiver. All contrast images of TAD mode were acquired with the same instrument parameters (Mechanical Index (MI) = 0.13, gain (Gn) = 24 dB, depth = 8 cm). Through the image analysis with the instrument software, the time intensity curves, mean video intensity of DiR-SPIO-NDs and SonoVue in 25 seconds were obtained.

### 6. *In vivo* Imaging Study

#### Ethics statement

All animal experiments were approved by the Institutional Animal Care and Use Committee of College of Life Science and Technology at Huazhong University of Science and Technology.

#### 6.1. In vivo fluorescence imaging

The BALB/c nude mice (male, 4–6 weeks, 18–23 g) were injected with 0.2 mL of DiR-SPIO-NDs (5.6×10^7^ nanobubbles/mL) at a dose of 0.072 mg DiR/kg body weight *via* tail vein, and anesthetized with 1.5% of isoflurane/oxygen. Then the anesthetized mice in the ventral positions were put into the chamber of *in vivo* image system (IVIS, Lumina XR, Caliper Life Sciences, USA) and the post-injection fluorescence images were obtained at 10 min, 1 h, 2 h, 3 h, 4 h, 6 h, 8 h, 10 h and 29 h, respectively. During the imaging process, the 1% of isoflurane/oxygen anesthesia was delivered *via* the nose cone system to maintain the anesthesia. All images were acquired with the following parameters: Exposure time = auto, binning = 4, f/stop = 2, FOV = 10 cm. Filter sets were fixed with the following parameters: excitation wavelengths was 745 nm and emission filter was set at ICG. Acquired images were processed and analyzed by using Living Imaging software (Version 4.2, Caliper Life Sciences).

After *in vivo* fluorescence imaging, the mice were sacrificed at 30 h post injection. The major organs such as heart, liver, spleen, lung and kidney were removed, and the *ex vivo* fluorescent images of the organs were also acquired and analyzed with the same system as described above.

#### 6.2. In vivo MRI

SD rats (male, 180–200 g) were anaesthetized by 10% of trichloroacetaldehyde hydrate with a dose of 0.4 mL per 100 g body weight. Then the rats were placed in a 3T whole-body MR scanner combined with an 8-channel wrist joint coil and scanned before and after administration of 1 mL of DiR-SPIO-NDs with a dose of 1.5 mg Fe/kg body weight through the tail vein for 7 h. The scanning parameters were as the following: Field of view (FOV) = 100 mm, averages = 4, base resolution = 192×192, slice thickness = 3 mm, echo time (TE) = 62 ms, repetition time (TR) = 3000 ms, flip angle = 120°, and scanning time = 2–3 min. The coronal and axial planes with T_2_-weighted imaging were acquired on the MR scanner. The signal intensities (SI) of liver, spleen, kidney and muscle of rats were obtained at the relatively homogeneous region of interest (ROI) with same diameters in the same slice on T_2_-weighted imaging at each time point. The relative signal enhancement values (RSEs) were plotted against time. The RSEs were calculated by using the following formula:
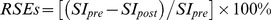
where SI_pre_ and SI_post_ were the signal intensity of rat organs before and after the administration of DiR-SPIO-NDs, respectively.

Histology analysis of the rat organs was done at 8 h after injection of DiR-SPIO-NDs. The rats were sacrificed and perfused with normal saline (250 mL) and 4% of paraform (250 mL). The liver, spleen, kidney and lung of rats were removed and soaked into 4% of paraform for at least 24 h. Then the paraform fixed species were embedded in paraffin and sliced. The tissue sections were treated with Prussian blue staining and nuclear fast red solution counterstaining per standard clinical laboratory protocol.

#### 6.3. In vivo ultrasound imaging

Human hepatoma cells HepG2 were implanted into the BALB/c nude mice (male, 4–6 weeks, 18–23 g) to grow tumor xenografts in this study. Briefly, the cells were re-suspended in 0.1 mL of PBS solution and subcutaneously injected into the right lower back of unanaesthetized mice (5×10^7^ cells/mouse). When the tumor volume reached to 400–450 mm^3^ (about 6 weeks later), the experiments were performed *in vivo* by using a 9L linear transducer in the clinical US scanner system (Logiq9, GE, USA). 50 µL of DiR-SPIO-NDs were injected intratumorally (1.2×10^8^ nanobubbles/mL), and the ultrasound transducer was simultaneously placed at the tumor region to investigate the contrast enhancement at the time points of 0 min, 1 min and 4.5 h, respectively. The space between the transducer and the tumor was filled with adequate ultrasonic transmission gel. Ultrasonic images were acquired by using the 8.0 MHz transducer in B–mode, and the scanning parameter setting were optimized at the beginning of the experiment (MI = 0.13, Gn = 36 dB, depth = 2 cm) and unchanged during the entire imaging session.

## Results and Discussion

### 1. Fabrication of DiR-SPIO-NDs

**Figure 2:**
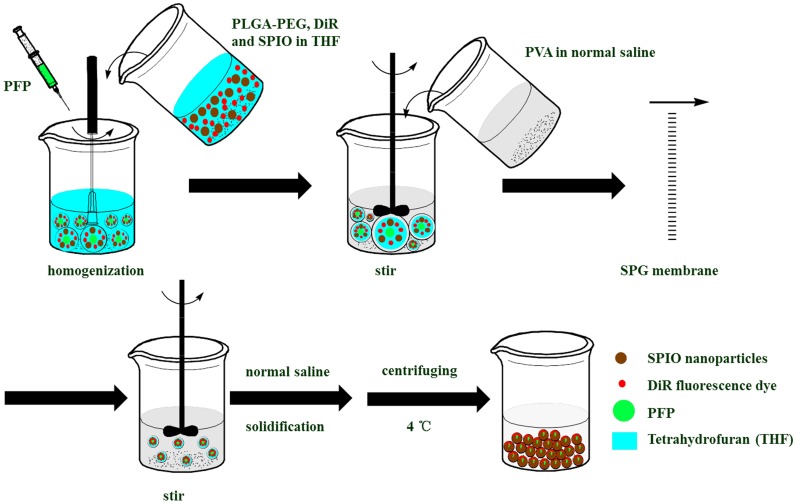
Stepwise fabrication of DiR-SPIO nanodroplets suspension.

The design and fabrication of nanodroplets were illustrated in Figure 2. Firstly, the gas-in-oil primary emulsions were formed with the PFP as gas phase and THF solution (including PLGA-PEG-PLGA, DiR and SPIO nanoparticles) as oil phase through homogenization in the ice bath and in the dark. Then, the gas-in-oil-in-water coarse double emulsions with PVA as the outer water phase were formed from the above primary emulsions by stirring mechanically. Next, to further improve the stability and narrow the size distribution, the obtained gas-in-oil-in-water premix double emulsions were repeatedly extruded through the uniform pores of a SPG membrane under high pressure until getting a liquid jet. At this moment, the shear force inside SPG membrane channels was very strong due to the tortuosity and constriction of the membrane channels and branches. Therefore, the obtained emulsion droplet size was smaller than the membrane pore size and turned to uniform after extruding through the membrane for five times. Note that the droplets were not deformed because the final droplet size was smaller than the pore size [17], [18]. Finally, THF was evaporated and the droplets were solidified to form nanodroplets under stirring. Because the PFP was liquid at 4°C and the density was larger than that of water, the nanodroplets were concentrated to a certain concentration by centrifuging at 4°C for the following experiments.

Thus, by combining SPG membrane emulsification with double emulsions solvent evaporation, we developed a novel method for preparation of vehicle acoustic nanodroplets. Unlike the conventional sonication and emulsion-evaporation methods, which were usually resulting in instable and a relatively broad size distribution, the as-developed nanodroplets were stable and uniform. This could be explained by the fact that the liquid PFP (boiling point 29°C) was stable during preparation and the nanodroplets were difficult to break.

### 2. Characterization of DiR-SPIO-NDs

**Figure 3:**
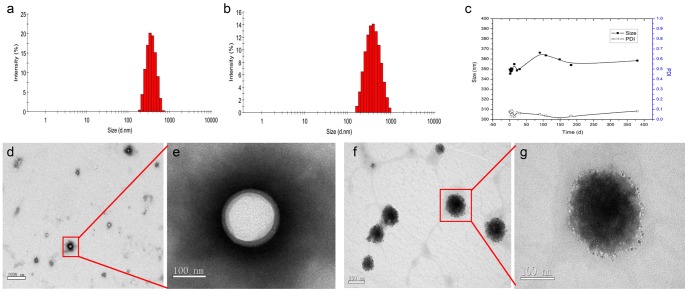
The size distribution and morphology of nanodroplets. Size distributions of (a) NDs and (b) DiR-SPIO-NDs. (c) Stability of NDs in normal saline. (d) TEM image of nanodroplets without fluorescence dye DiR and SPIO nanoparticles (bar = 1000 nm), (f) TEM image of DiR-SPIO-NDs (bar = 200 nm), and (e) and (g) are the magnified views (bar = 100 nm) of a portion (red frame) of (d) and (f), respectively.

Figure 3a and b showed that the hydrodynamic sizes of DiR-SPIO-NDs and NDs at 37°C were 385.0±5.0 nm and 348.7±2.3 nm with PDI of 0.169±0.011 and 0.072±0.010, respectively. Meanwhile, the zeta potential were 11.40±1.51 mV and –3.36±0.43 mV, respectively. The results indicated that the size of NDs was enlarged, size distribution was broadened and the surface charge was changed from negative to positive when SPIO and DiR were included.

Because stability of NDs is crucial to their application as contrast agents, the size and PDI of NDs were evaluated in normal saline for one year by DLS. As shown in Figure 3c, the relative standard deviation (RSD) of size was less than 1.8% and PDI was less than 0.1 through one year. These results indicated the NDs were highly stable in normal saline at 4°C. In contrast, the commercially available microbubbles SonoVue only remained 6 hours. This could be attributed to stabilizing polymer shells and low diffusivity of PFP gas, which increased the stability of NDs [28].

[Fig pone-0085003-g004] Figure 5e and f showed the microscopy images of DiR-SPIO-NBs at bright and fluorescence field after NBs being and sonicated for 2 min at 37°C. When the microscopy image was magnified by 400 folds, obvious hollow circles were observed with uniform distribution at bright field. Accordingly, the corresponding images were captured at fluorescence field. In order to acquire further detailed morphology of NDs, TEM image was obtained. Figure 3d was image of nanodroplets without fluorescence dye DiR and SPIO nanoparticles, the interface between the shell and gas could be discerned (Figure 3e). The results showed that NDs were core-shell spherical nanoparticles with a certain thickness of shell. After assembling with DiR and SPIO, the previous core-shell structure became solid with SPIO nanoparticle clusters in the polymer shell and DiR fluorescence dyes in the entire nanodroplets (Figure 3f and g). The mean diameter of DiR-SPIO-NDs based on TEM images was approximately 200 nm, which was smaller than that of about 385 nm determined by DLS, arising from the aqueous state of the NDs [29].

**Figure 4 pone-0085003-g004:**
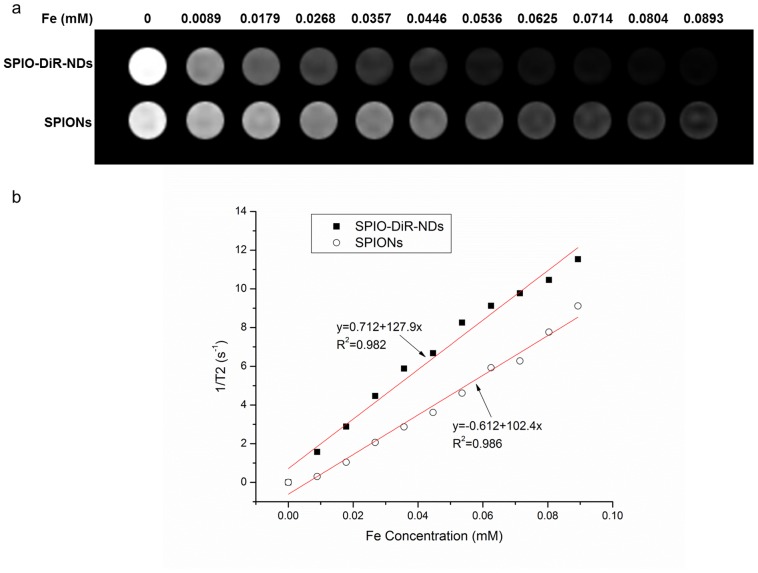
Magnetic properties of DiR-SPIO-NDs and hydrophilic SPIO nanoparticles. (a) T_2_-weighted MR images; (b) T_2_ relaxation rate (1/T_2_, s^−1^).

**Figure 5:**
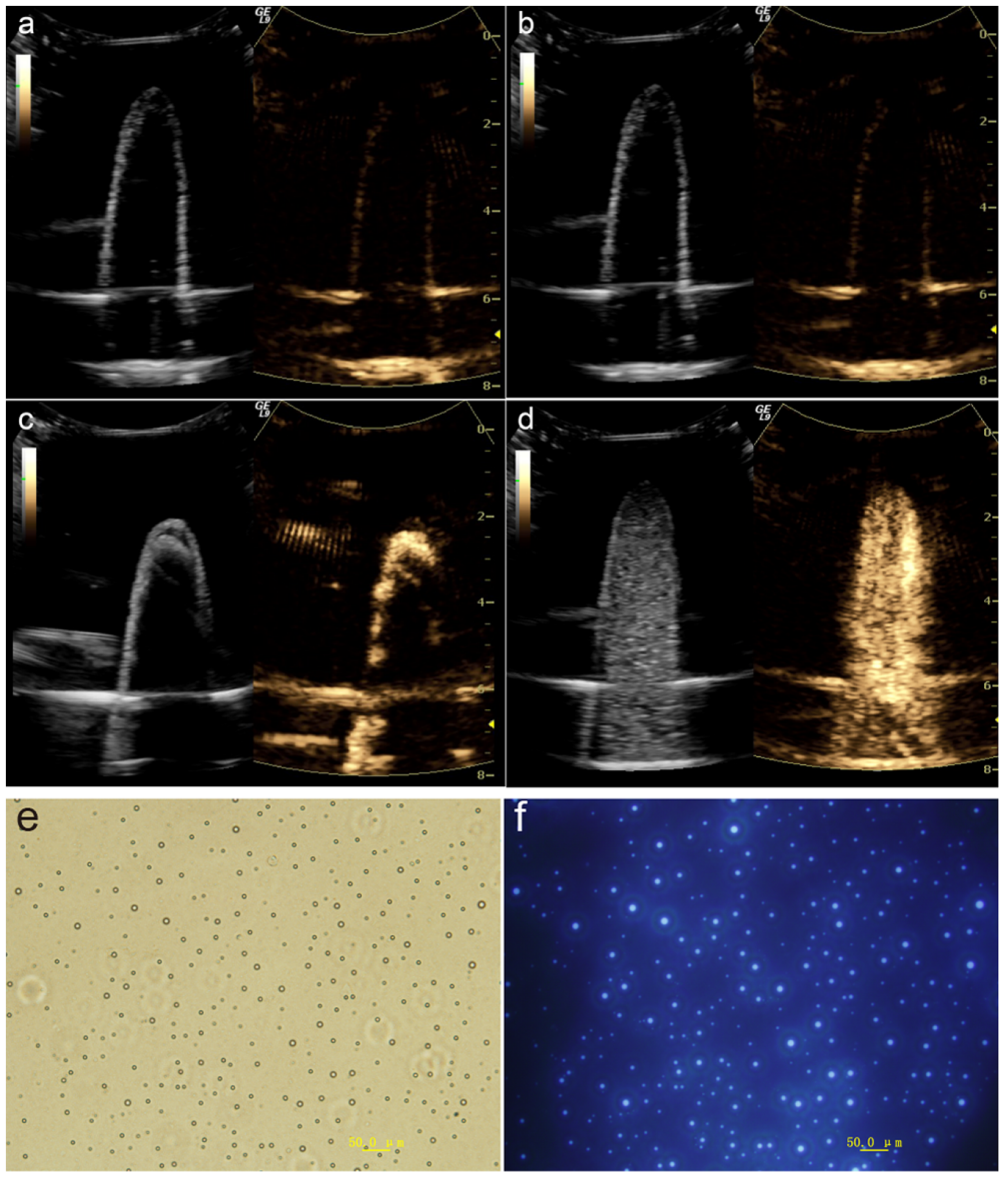
Ultrasound-triggered phase transition of DiR-SPIO-NDs *in vitro*. (a–d) The ultrasound imaging of the different samples *in vitro* by using 3.5 MHz ultrasound transducer in TAD mode: (a) Before injection of samples, and after injection of (b) normal saline, (c) SonoVue, (d) DiR-SPIO-NDs; the left was B-mode image and the right was the corresponding contrast image in each picture. (e–f) Microscopy images (400×) of diluent DiR-SPIO-NBs after sonication for 2 min at 37°C. (e) Bright field, (f) fluorescence field.

The total iron content in DiR-SPIO-NDs was 319.3 mg/L. The concentration of DiR-SPIO-NBs was about 2.8×10^9^ nanobubbles/mL by counting on a hemacytometer. Because nanobubbles or smaller microbubbles could not be detected, the actual concentration of nanobubbles in the experiment might be higher.

Both microbubbles and SPIO nanoparticles (SPIONs) could shorten the transverse relaxation time in MRI, and SPIONs had been used as a negative contrast agent [7], [30], therefore, the ability of DiR-SPIO-NDs to enhance magnetic resonance imaging was evaluated. T_2_-weighted MR images for different iron concentrations of nanodroplets suspension were acquired on the clinical 3T MRI instrument. As shown Figure 4a, in the MR images of the DiR-SPIO-NDs and hydrophilic SPIO nanoparticles, the sensitive and concentration-dependent dark areas (dark signal) were both observed. And DiR-SPIO-NDs could reduce the transverse relaxation time T_2_ more significantly compared with the hydrophilic SPIO nanoparticles at the same concentration. The results might be due to the fact that nanodroplets/nanobubbles made the SPIO nanoparticles in the shell form a similar cluster of SPIO with the same SPIO concentration, which then made the number of SPIO per volume higher than that of free hydrophilic SPIO nanoparticles anywhere else.

As expected, the R_2_ relaxation rate (1/T_2_) was highly linearly proportional to the iron concentration (Figure 4b) with the following relationship [7], [29]:
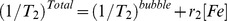
where (1/T_2_)^Total^ was the observed overall relaxation rate in the presence of SPIO nanoparticles, (1/T_2_)^bubble^ was the relaxation rate of nanodroplets/nanobubbles, [Fe] was the concentration of SPIO nanoparticles, r_2_ was the transverse relaxivity, and r_2_ [Fe] was the relaxation rate of SPIO nanoparticles.

Generally, r_2_ [Fe]>>(1/T_2_)^bubble^. Therefore, the data of the DiR-SPIO-NDs were fitted to a linear function nicely with square of the correlation coefficient R^2^ = 0.982. The calculated R_2_ relaxivity of the DiR-SPIO-NDs and hydrophilic SPIO nanoparticles were 127.9 mM^−1^s^−1^ and 102.4 mM^−1^s^−1^, respectively. These results suggested that the DiR-SPIO-NDs could be used as a negative MRI contrast agent in T_2_-weighted MR imaging.

### 3. Ultrasound-Triggered Phase Transition of DiR-SPIO-NDs

Although the boiling point (29°C) of PFP at atmospheric pressure was lower than physiological temperatures (37°C), the superheated PFP was still maintained as liquid in the polymer shell at 37°C within a short time. And the phase transition from droplets to bubbles with evaporation didn’t happen until the droplets were activated by ultrasound [24]. To investigate the ultrasound-triggered phase transition of DiR-SPIO-NDs, we studied the *in vitro* ultrasound images of the DiR-SPIO-NDs, and compared with that of SonoVue and normal saline. When the samples were irradiated by the clinical 3.5 MHz ultrasound transducer at 37°C in a water bath, compared with pre-injection (Figure 5a) and the degassed normal saline (Figure 5b), DiR-SPIO-NDs (Figure 5d) were brighter in the latex finger cot in B-mode image and distributed in the whole space of latex finger cot. Additionally, the color-coded spots reflecting the enhancement of echogenic movement signals also appeared in the corresponding contrast imaging. These results suggested that the PFP core in the polymer shell could be stimulated to transform from a liquid to a gas under the ultrasound irradiation at physiological temperature, thereby the DiR-SPIO-NDs droplets could be converted into larger nanobubbles or microbubbles to obtain ultrasound imaging. The SonoVue microbubbles (Figure 5c) presented similar results. But it only became cloudier at the beginning and then aggregated quickly. In the *in vitro* ultrasound imaging, the mean video intensities of DiR-SPIO-NDs and SonoVue (the mean diameter 2.5 µm) in 25 seconds were 19.18 dB and 24.16 dB, respectively. These results indicated that the imaging intensity of nanobubbles was weaker than that of microbubbles under the clinical diagnostic ultrasound conditions, but microbubbles aggregated and were destroyed more easily by acoustic cavitations. Therefore, DiR-SPIO-NBs were more stable compared with SonoVue under the clinical diagnostic ultrasound conditions.

Furthermore, the inverted fluorescence microscopy images (Figure 5e and f) of DiR-SPIO-NBs were observed. The DiR-SPIO-NDs could not be observed on an inverted fluorescence microscopy when they were in small droplets. However, when the DiR-SPIO-NDs were insonated at 37°C for 2 min, the microscopy image could be observed distinctly because the droplets were vaporized to form larger nanobubbles and microbubbles.

### 4. Biocompatibility of DiR-SPIO-NDs

**Figure 6:**
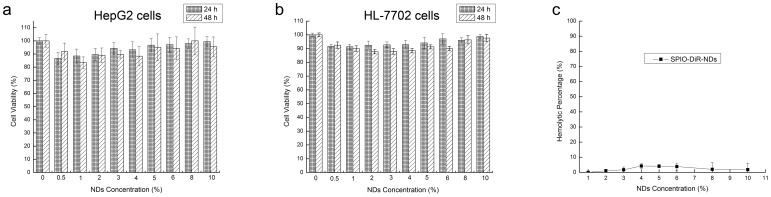
Biocompatibility of DiR-SPIO-NDs. MTT assay results for (a) HepG2 cells and (b) HL-7702 cells after incubation 24 h and 48 h. (c) Hemolysis assay results. Data were expressed as mean ± SD (n = 5).

The biocompatibility of NDs was evaluated by using two independent methods [13]. In order to investigate the cytotoxicity of the DiR-SPIO-NDs, tumor cells (HepG2 cells) and normal cells (HL-7702 cells) were used to evaluate the cell viability *via* MTT assay. Figure 6a and b showed the cells viability after 24 h and 48 h of incubation with different concentrations of DiR-SPIO-NDs. A viability of over 80% in the two cell lines was maintained by increasing the NDs concentration from 1.4×10^7^ to 2.8×10^8^ nanobubbles/mL (from 0.5% to 10% of commonly used NDs concentrations). The results indicated that the DiR-SPIO-NDs had no obvious cytotoxicity to HepG2 and HL-7702 cells within the concentration range used during the *in vivo* imaging.

In order to evaluate the safety of DiR-SPIO-NDs, their hemolytic activities were analyzed. As shown in Figure 6c, less than 5% of hemolysis was maintained at all evaluated concentrations, with double-distilled water as a positive control having 100% of hemolysis under the experimental conditions. The results indicated that the DiR-SPIO-NDs would be anhemolytic toward erythrocytes after the intravenous injection. Thus, the cytotoxicity and hemolysis studies strongly suggested that the DiR-SPIO-NDs had good biocompatibility and suitability for biomedical applications.

### 5. *In vivo* Imaging Studies of DiR-SPIO-NDs

#### 5.1. Fluorescence imaging

**Figure 7:**
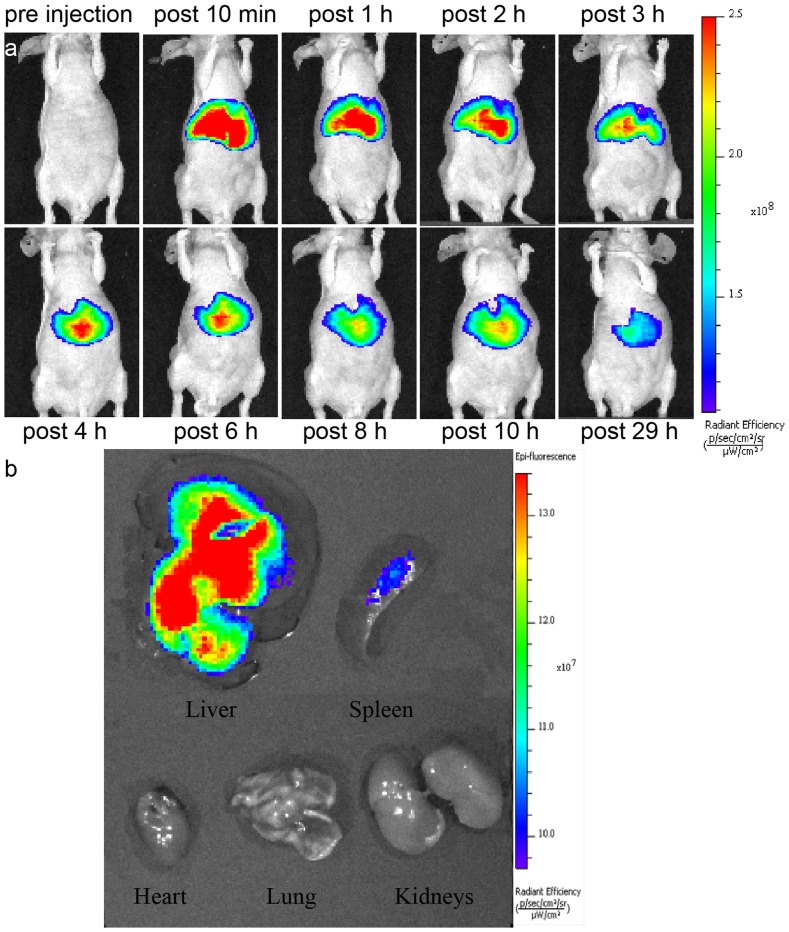
*In vivo* fluorescence imaging in nude mice before and after intravenous injection of DiR-SPIO-NDs. (a) Time-dependent images of DiR-SPIO-NDs in mice ventral views, (b) *ex vivo* images of organs harvested from the mice at 30 h after injection.

In this study, the biodistribution of DiR-SPIO-NDs *in vivo* was evaluated by using a noninvasive near infrared optical imaging technique [31]. Figure 7a showed the real-time images of DiR-SPIO-NDs *in vivo* in nude mice. After DiR-SPIO-NDs were injected *via* the tail vein, time-dependent biodistribution was observed. The strongest fluorescence signals were detected in most organs of the mice within 10 min, which was resulted from the circulation of nanodroplets in the bloodstream. Additionally, the fluorescence intensity in the liver tissue was much higher and maintained at a high level up to 29 h. The *ex vivo* fluorescence evaluation of major organs at 30 h after the injection also revealed that the accumulation of DiR-SPIO-NDs in liver was much more than that in other organs, followed by that in spleen (Figure 7b), probably because DiR-SPIO-NDs were mainly captured by macrophages of the reticuloendothelial system (RES) in the liver and spleen. These observations indicated that DiR-SPIO-NDs were highly selectively accumulated and had a prolonged maintaining time in liver. Therefore, DiR-SPIO-NDs demonstrated a great potential in the fluorescence imaging of liver.

#### 5.2. MRI

**Figure 8:**
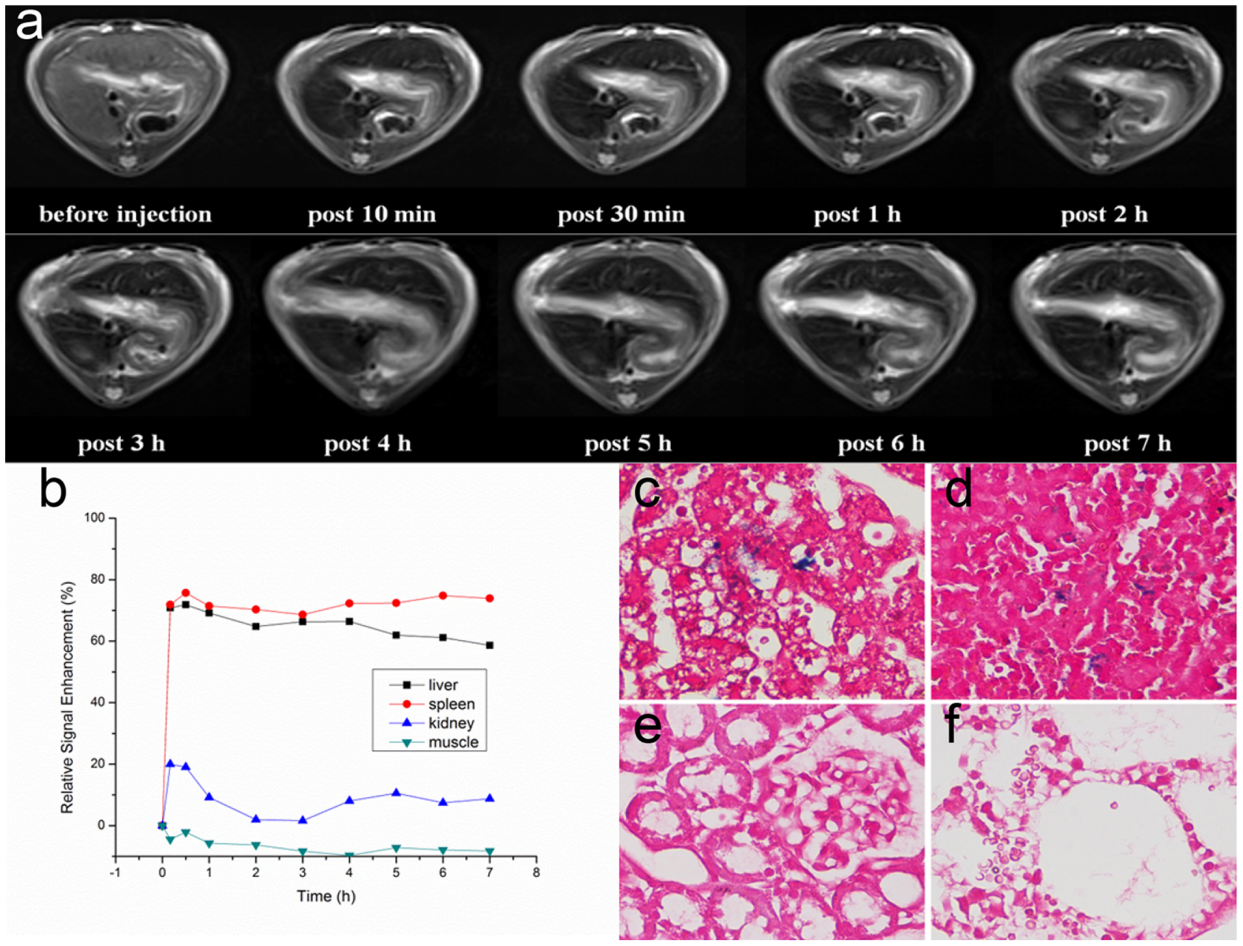
*In vivo* MRI in SD rat before and after intravenous injection of DiR-SPIO-NDs. (a) T_2_-weighted images of rats at different time points before and after tail vein administration of DiR-SPIO-NDs; (b) Relative signal enhancement values in liver, spleen, kidney, and muscle before and after the injection of DiR-SPIO-NDs. (c–f) Prussian blue stained images (400×) of (c) liver, (d) spleen, (e) kidney and (f) lung after 8 h with the tail vein injection of DiR-SPIO-NDs.

The DiR-SPIO-NDs with higher R_2_ relaxivity were expected to enhance the MRI and to ease the toxicity with a decreased dose [32]. In general, once injected intravenously, the MRI contrast agents including magnetic nanoparticles were easily accumulated in liver and spleen tissues (e.g. commercially available Resovit and Feridex), which was beneficial to the MR imaging and diagnosis of liver and spleen disease [33]. To evaluate the feasibility of DiR-SPIO-NDs as MRI contrast agent *in vivo*, male SD rat models were used. Figure 8a showed the T_2_-weighted MR images of a typical rat liver at different time points after the injection of DiR-SPIO-NDs *via* tail vein. It could be found that, with the DiR-SPIO-NDs injection, the overall liver region was darkened significantly after 10 min compared with that before injection, and a high contrast of liver tissue persisted within 7 h. This observation suggested the DiR-SPIO-NDs demonstrated superior MRI capability on liver.

The time courses of T_2_ relative signal enhancement in liver, spleen, kidney and muscle after the DiR-SPIO-NDs injection were shown in Figure 8b. Consistent with the findings of fluorescence imaging of DiR-SPIO-NDs, the results of MRI *in vivo* verified the DiR-SPIO-NDs distribution as well. With the injection of DiR-SPIO-NDs, both the liver and spleen contrast were enhanced significantly after 10 min and RSEs were greater than 70%. The highest contrast enhancement of liver was lasted for 4 h, and then gradually decreased to about 58% after 7 h. The highest contrast enhancement of spleen sustained for 7 h without significant change. These results suggested the DiR-SPIO-NDs might be ingested by the macrophages of RES in liver and spleen. The highest RSEs of kidney was achieved to about 20% after 10 min and then gradually decreased to less than 10% over a period of 1 h, which indicated that DiR-SPIO-NDs could be excreted slowly through the kidney. Furthermore, the RSEs of muscle had no significant change within 7 h, which confirmed the DiR-SPIO-NDs could not permeate into other normal tissues and they were safe for other organs.

To further confirm the DiR-SPIO-NDs uptake by the liver and spleen tissues, Prussian blue staining of slides from different organs, including the liver, spleen, kidney and lung, were obtained. The presence of the blue color in the images indicated the accumulation of DiR-SPIO-NDs in the organs. As shown in Figure 8c and d, some parts of liver and spleen were stained blue color, which suggested the presence of iron. In contrast, the images of kidney and lung did not show significant Prussian blue staining (Figure 8e and f). Therefore, it was clear that the DiR-SPIO-NDs produced MRI contrast enhancement primary in liver and spleen. These results were consistent with the previous findings obtained from fluorescence imaging and MR imaging.

#### 5.3. US imaging

**Figure 9:**
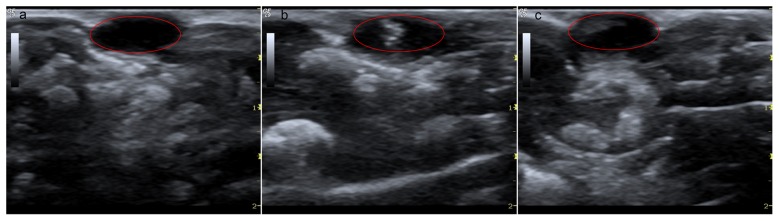
*In vivo* B-mode ultrasound images of subcutaneous tumor with injection of DiR-SPIO-NDs. Ultrasound images were obtained with Logiq9 system with a 9L linear transducer at 8.0(a) Before the injection, (b) 1 min after injection, (c) 4.5 h after injection. The red circle denoted the echo enhancement in the tumor region.

In order to evaluate the ultrasound imaging capability of the DiR-SPIO-NDs as ultrasound contrast agent *in vivo*, the nude mice bearing HepG2 tumor models following intratumoral injection of nanodroplets were used [34]. Figure 9 showed the B-mode ultrasound imaging of tumor before the injection and after both 1 min and 4.5 h of the injection. The red circle range denoted the tumor region. The dark hypoechoic area could be found in the tumor before the DiR-SPIO-NDs injection (Figure 9a). After 1 min with injection, the strong hyperechoic bright spots were generated in the tumor (Figure 9b). And they persisted for at least 4.5 h, although the brightness was gradually decreased (Figure 9c). It was well-known that nanobubbles usually had weaker ultrasound contrast ability than mirobubbles and ultrasound imaging of small size nanobubbles under clinical diagnostic ultrasound was difficult [35]. However, the ultrasound image of DiR-SPIO-NDs can not only be available but also persist for a long period in the experiment, which might be attributed to the phase transition of DiR-SPIO-NDs from droplets to bubbles under clinical ultrasound irradiation within one minute, thus the nanobubbles would induce volumetric expansion and transform to microbubbles for the significant enhancement of ultrasound contrast. In addition, the low-diffusivity-gas core materials PFP and rigid biodegradable polymer shell stabilized the microbubbles *in vivo*, thus resulting in a long-lasting ultrasound contrast. These results suggested that DiR-SPIO-NDs potentially could become a stable enough and long-lasting contrast agent in the clinical ultrasound imaging.

## Conclusions

The vehicle acoustic nanodroplets DiR-SPIO-NDs with ultrasound-triggered phase transition were prepared by premix SPG membrane emulsification method. The nanodroplets were composed of a liquid PFP core and a polymer shell. The core-shell spherical shape was confirmed by TEM and microscopy. And the fluorescence dye DiR and SPIO nanoparticles were dispersed in the shell or core of DiR-SPIO-NDs. The nanodroplets showed high stability in normal saline at 4°C for one year. The MTT assay and hemolysis studies demonstrated that the nanodroplets were biocompatible. Furthermore, the specific distensible property of NDs was confirmed by ultrasound imaging *in vitro*. The MRI and fluorescence imaging showed that the DiR-SPIO-NDs had high R_2_ relaxivity and good enhanced contrast effects in liver. The ultrasound imaging in tumor revealed the DiR-SPIO-NDs had long-lasting contrast ability for clinical diagnostic ultrasound. In addition, the results *in vitro* and *in vivo* also suggested that both SPIO nanoparticles and DiR fluorescence dye were well encapsulated in the nanodroplets and the DiR-SPIO-NDs could be potentially used as a MRI/US/fluorescence multimodal imaging contrast agent in the diagnosis of liver tissue diseases.

## Supporting Information

Figure S1 **Striking image of the designed ultrasound-triggered phase transition DiR-SPIO-NDs with multimodal imaging functionality.** The uniform-sized stable nanodroplets were prepared by SPG membrane emulsification method. Under ultrasound irradiation at 37°C, nanodroplets transformed to microbubbles for MRI/fluorescent/ultrasound tri-modal imaging in the diagnosis of liver tissue diseases.
(TIF)
