# Supplementary figures and images for: Ultrasound-Triggered Phase Transition Sensitive Magnetic Fluorescent Nanodroplets as a Multimodal Imaging Contrast Agent in Rat and Mouse Model
Source: PLoS One. 2013 Dec 31;8(12):e85003. doi: 10.1371/journal.pone.0085003 (PMC3877337; doi:10.1371/journal.pone.0085003)

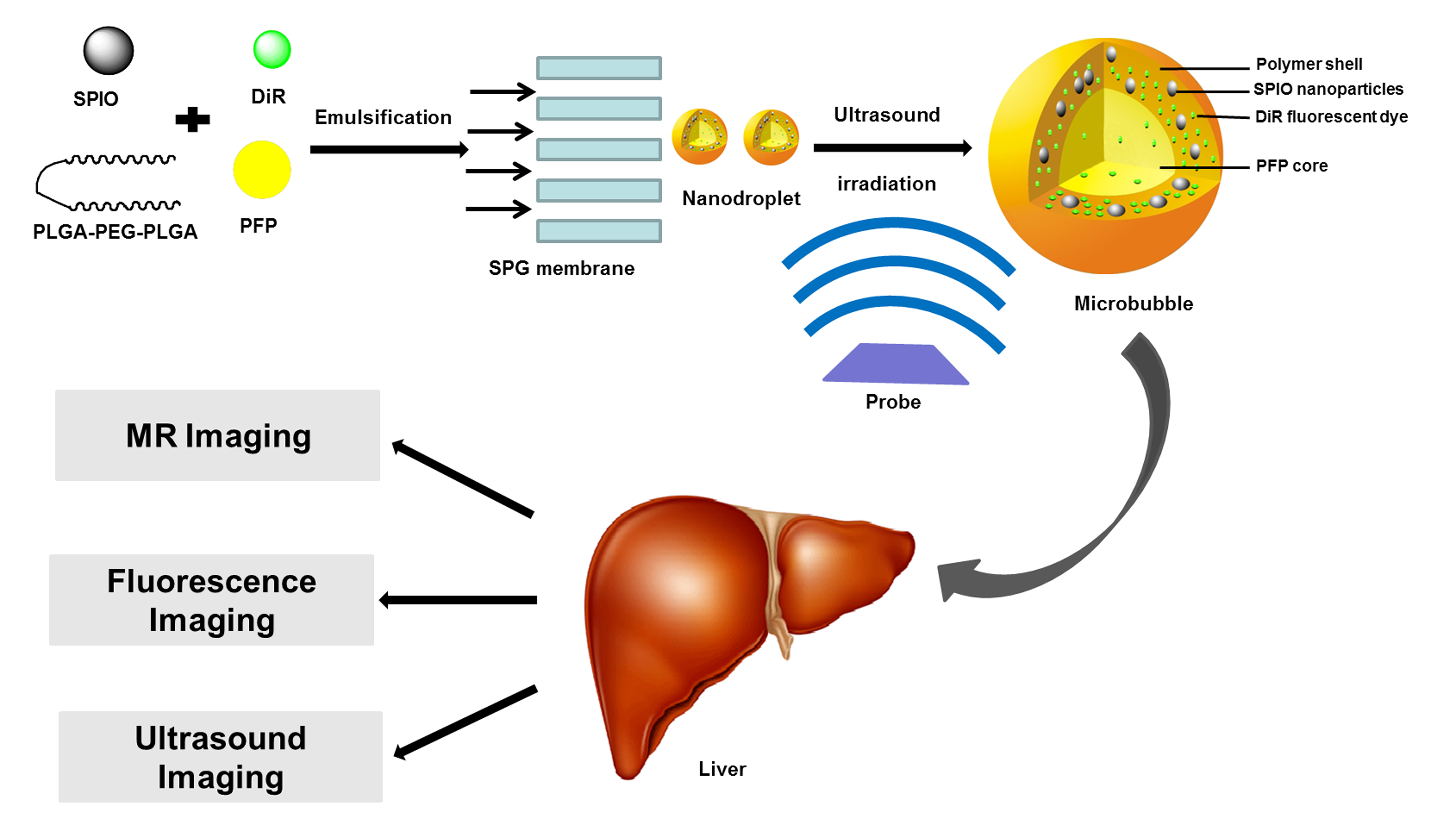

Supplement: Figure S1 — Striking image of the designed ultrasound-triggered phase transition DiR-SPIO-NDs with multimodal imaging functionality. The uniform-sized stable nanodroplets were prepared by SPG membrane emulsification method. Under ultrasound irradiation at 37°C, nanodroplets transformed to microbubbles for MRI/fluorescent/ultrasound tri-modal imaging in the diagnosis of liver tissue diseases. (TIF) [file pone.0085003.s001.tif]
